# Supplementary material for: Poly(ester imide)s Possessing Low Coefficients of Thermal Expansion and Low Water Absorption (V). Effects of Ester-linked Diamines with Different Lengths and Substituents
Source: Polymers (Basel). 2020 Apr 8;12(4):859. doi: 10.3390/polym12040859 (PMC7240679; doi:10.3390/polym12040859)
Supplement: Supplementary file 1 [file polymers-12-00859-s001.pdf]

Supporting Information 1

SI 1. The abbreviations, commercial sources, and melting points of the raw materials used in this study.

Raw materials	Source	Melting point (°C)
Hydroquinone (HQ)	Wako Chemical	173 [a]
Methylhydroquinone (MHQ)	Tokyo Chemical Industry (TCI)	126 [b]
Methoxyhydroquinone (MeOHQ)	TCI	89 [a]
Phenylhydroquinone (PHQ)	Aldrich	100 [b]
1,4-Dihydroxynaphthalene (14DHN)	TCI	
Resorcinol (RC)	TCI	111 [b]
4,4′-Biphenol (44BP)	TCI	287 [a]
3,3′-Diphenyl-4,4′-biphenol (DP44BP)	Honshu Chemical Industry	149 [a]
MHQHB	Home-made	318 [a]
4-Nitrobenzoyl chloride (4-NBC)	TCI	73 [b]

[a] Data determined from the endothermic peak measured at a heating rate of 5 °C min^{-1} on DSC.

[b] Data from the safety data sheet

Supporting Information 2

SI 2. The abbreviations, commercial sources, purification conditions, and melting points of the ester-linked diamines synthesized in this study.

Ester-linked diamines	Solvents for recrystallization	Vacuum-drying condition	Melting point [a] (°C)	Molecular formula (F_w, g/mol)	C, H, N (%) (Calcd.)	C, H, N (%) (Found)
AB-HQ	GBL	200 °C/12 h	294	$C_{20}H_{16}O_4N_2$ (348.36)	68.96, 4.63, 8.04	68.84, 4.50, 7.62
AB-MHQ	DOX	100 °C/12 h	275	$C_{21}H_{18}O_4N_2$ (362.38)	69.60, 5.01, 7.73	69.84, 4.98, 7.65
AB-MeOHQ	DOX	140 °C/12 h	254	$C_{21}H_{18}O_5N_2$ (378.38)	66.66, 4.79, 7.40	66.47, 4.77, 7.00
AB-PhHQ	DOX/CF (3/2, v/v)	110 °C/12 h	264	$C_{26}H_{20}O_4N_2$ (424.46)	-----	-----
AB-44BP	GBL	180 °C/12 h	-----	$C_{26}H_{20}O_4N_2$ (424.46)	73.57, 4.75, 6.60	73.41, 4.78, 5.95
AB-DP44BP	GBL	200 °C/12 h	323	$C_{38}H_{28}O_4N_2$ (576.65)	79.15, 4.89, 4.86	78.76, 4.98, 4.71
AB-14DHN	DMF + small quantity of EtOH	160 °C/12 h	274	$C_{24}H_{18}O_4N_2$ (398.42)	72.35, 4.55, 7.03	72.37, 4.60, 6.92

[a] Data determined from the endothermic peak measured at a heating rate of 5 °C min^{-1} on DSC. GBL =γ-Butyrolactone, DOX = 1,4-dioxane, CF = Chloroform, DMF = *N*,*N*-dimethylformamide.

Supporting Information 3

SI 3. The abbreviations, commercial sources, purification conditions, and melting points of the common monomers used in this study.

Common and ester-containing diamines	Source	Solvents for recrystallization	Vacuum-drying condition	Melting point [a] (°C)
**Diamines**				
4,4′-Oxydianiline (4,4′-ODA)	Wako Chemical	-----	50 °C/12 h	192
Bis(4-aminophenyl)terephthalate (BPTP)	Wakayama Seika	-----	50 °C/12 h	238
**Tetracarboxylic dianhydrides**				
Pyromellitic dianhydride (PMDA)	Mitsubishi Gas Chemical	-----	160 °C/24 h	286
3,3′,4,4′-Biphenyltetracarboxylic dianhydride (s-BPDA)	Tokyo Chemical Industry (TCI)	-----	200 °C/12 h	300
Hydroquinone bis(trimellitate) (TA-HQ)	Home-made	1,4-Dioxane	200 °C/12 h	277

[a] Data determined from the endothermic peak measured at a heating rate of 5 °C min^{-1} on DSC. DOX = 1,4-dioxane.

Supporting Information 4

Synthesis of AB-RC

AB-RC was synthesized by the reaction of resorcinol (RC) and 4-nitrobenzoyl chloride (4-NBC) in the presence of pyridine in anhydrous tetrahydrofuran (THF) and subsequent catalytic reduction of the dinitro compound obtained (m.p.: 189 °C) in *N*,*N*-dimethyformamide (DMF) at 80 °C for 4 h in a hydrogen atmosphere in the presence of Pd/C. The crude product was dissolved in a minimum quantity of hot DMF, followed by addition of a few drops of water to the hot DMF solution for inducing recrystallization. The precipitated black crystal was collected by filtration and dried at 120 °C for 12 h under vacuum. The analytical data of the product are as follows. Melting point (differential scanning calorimetry, DSC): 202 °C. FT-IR (KBr plate method, cm^{-1}): 3480, 3378, 3214 (amine, N–H), 1715 (ester, C=O), 1514 (1,4-phenylene). ^{1}H-NMR (400 MHz, DMSO-d_6, δ, ppm): 7.80 [d, 4H (relative integrated intensity: 4.42H), J = 8.6 Hz, 3,3′,5,5′-protons of the terminal aniline (AN) units], 7.48 [t, 1H (1.15H), J = 8.6 Hz, 5-proton of the central RC unit], 7.14–7.12 [m, 3H (3.45H), 2- + 4,6-protons of RC], 6.64 (d, 4H (4.59H), J = 8.7 Hz, 2,2′,6,6′-protons of AN], 6.20 [s, 4H (4.00H), amine]. The results confirm that the product is the desired diamine (AB-RC) shown below:

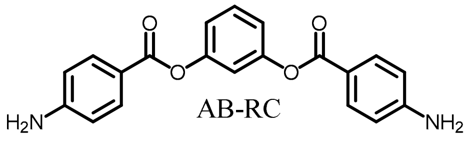

AB-RC

Supporting Information 5

Synthesis of AB-MHQHB

AB-MHQHB was synthesized by the reaction of an ester-linked bisphenol (MHQHB) and 4-NBC and subsequent catalytic reduction, according to the reaction schemes shown below.

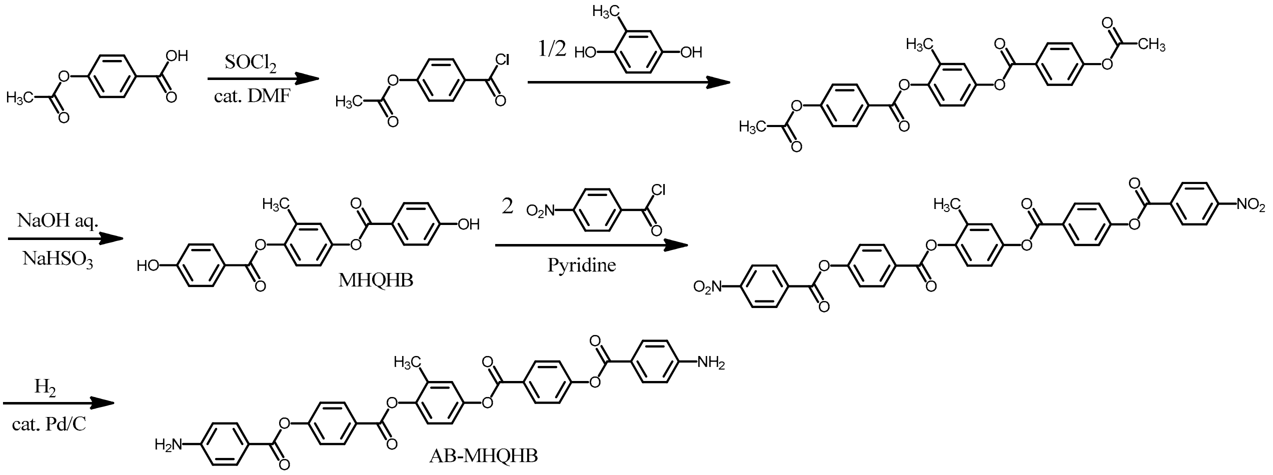

First, the ester-linked bisphenol (MHQHB) was synthesized and recrystallization from GBL. The analytical data is as follows. Melting point (DSC): 318 °C. FT-IR (KBr plate method, cm^{-1}): 3387 (O–H), 1699 (ester, C=O), 1512, 1495 (1,4-phenylene). ^{1}H-NMR spectrum (400 MHz, DMSO-d_6) of the product is shown below. The results confirm that the product is the desired bisphenol (MHQHB).

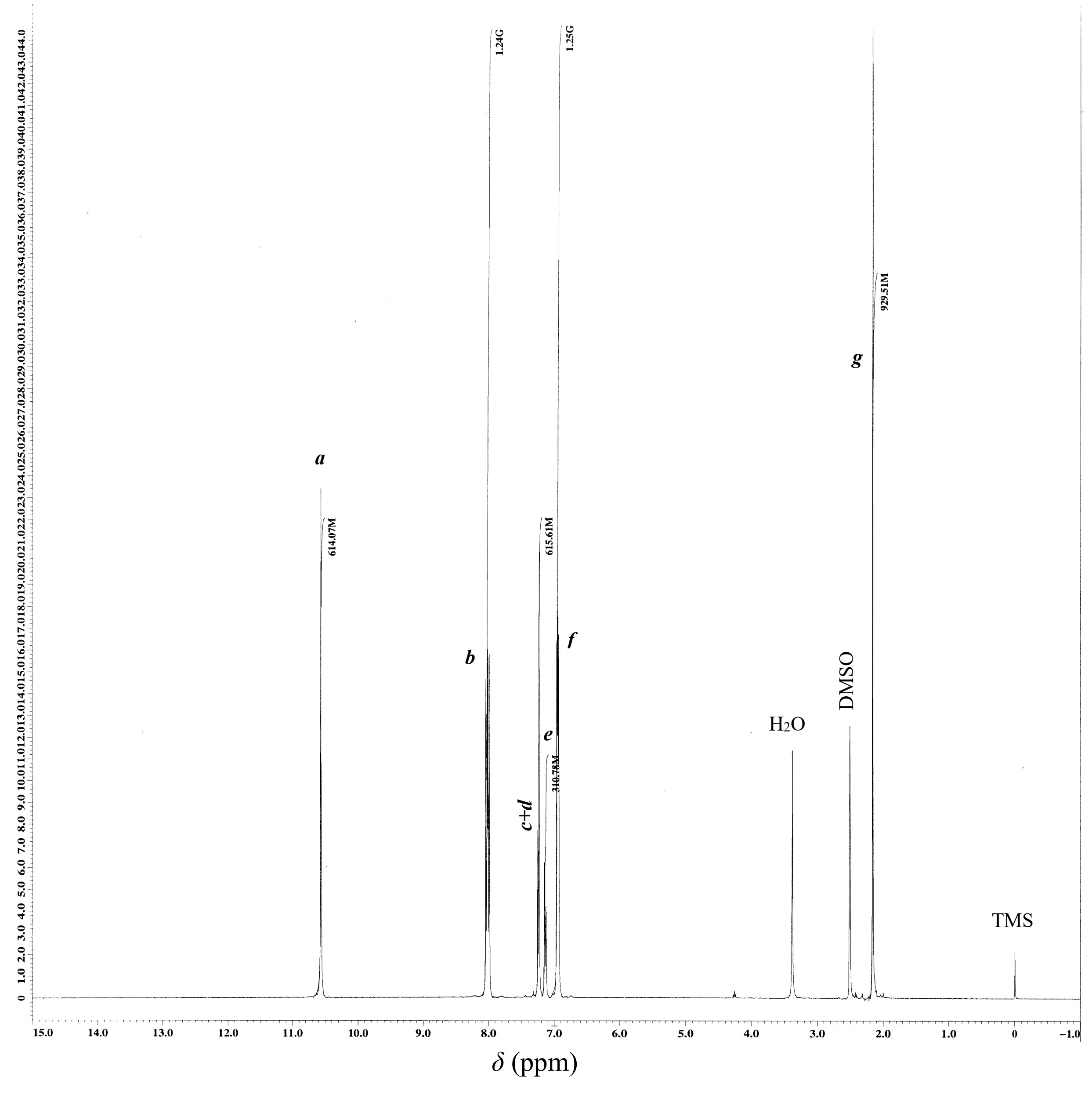
a
614.07M
b
1.24G
c+d
615.61M
e
310.78M
f
1.25G
H2O
DMSO
g
929.51M
TMS
15.0
14.0
13.0
12.0
11.0
10.0
9.0
8.0
7.0
6.0
5.0
4.0
3.0
2.0
1.0
0
−1.0
δ (ppm)

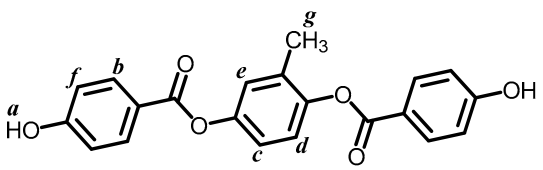
a
HO
f
b
O
O
e
g
CH3
c
d
O
O
OH

MHQHB

MHQHB was reacted with 4-NBC in the presence of pyridine in anhydrous DMF. The dinitro compound obtained was recrystallized from GBL (m.p.: 268 °C) and subsequently reduced in DMF at 100 °C for 4 h in a hydrogen atmosphere in the presence of Pd/C. The crude product obtained was recrystallized twice from GBL, and dried at 200 °C for 12 h under vacuum. The analytical data of the product are as follows. Melting point (DSC): 304 °C. FT-IR (KBr plate method, cm^{-1}): 3455, 3368 (amine, N–H), 1719 (ester, C=O), 1516 (1,4-phenylene). ^{1}H-NMR spectrum (400 MHz, DMSO-d_6) of the product is shown below. The results confirm that the product is the desired diamine (AB-MHQHB):

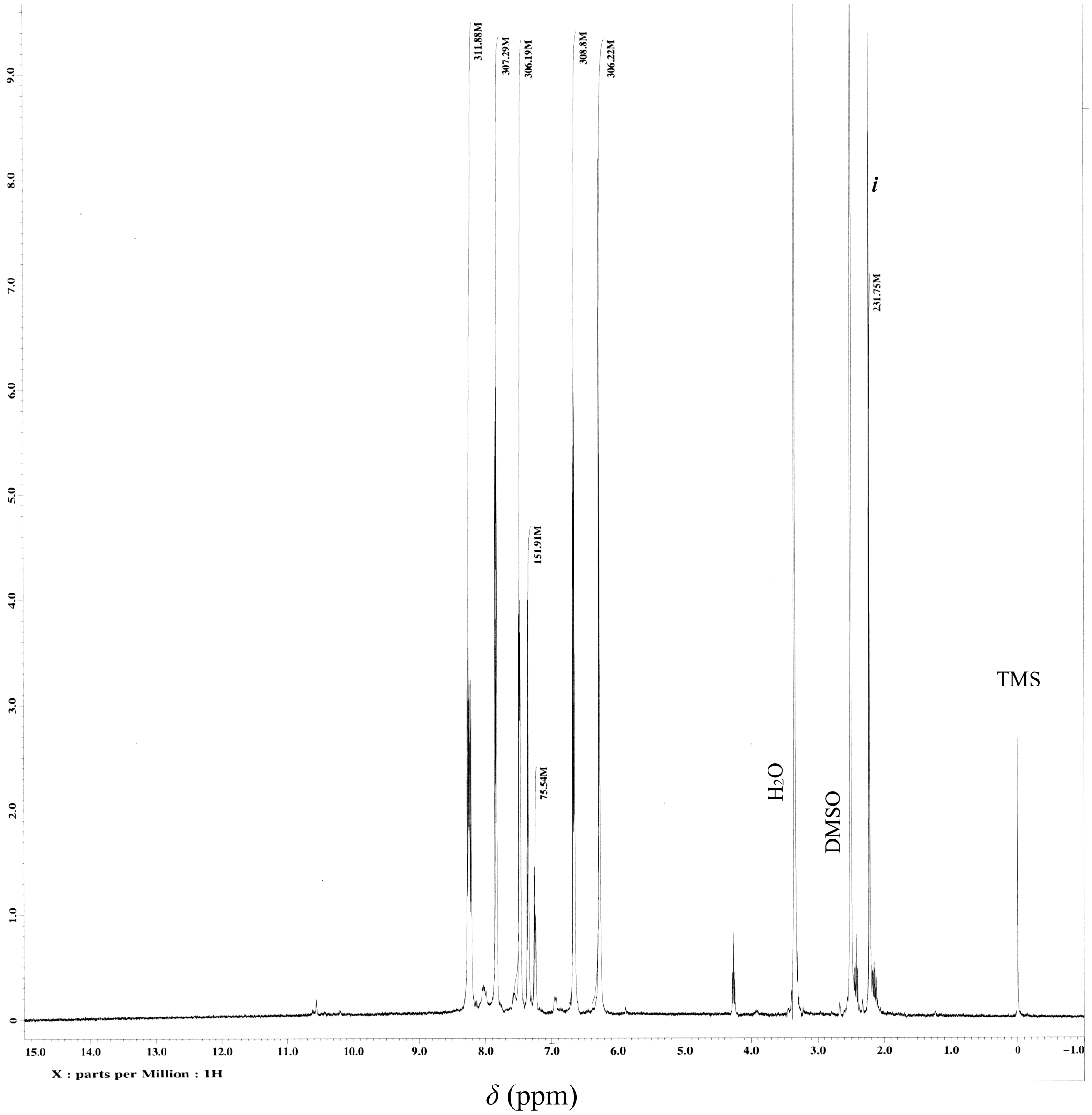

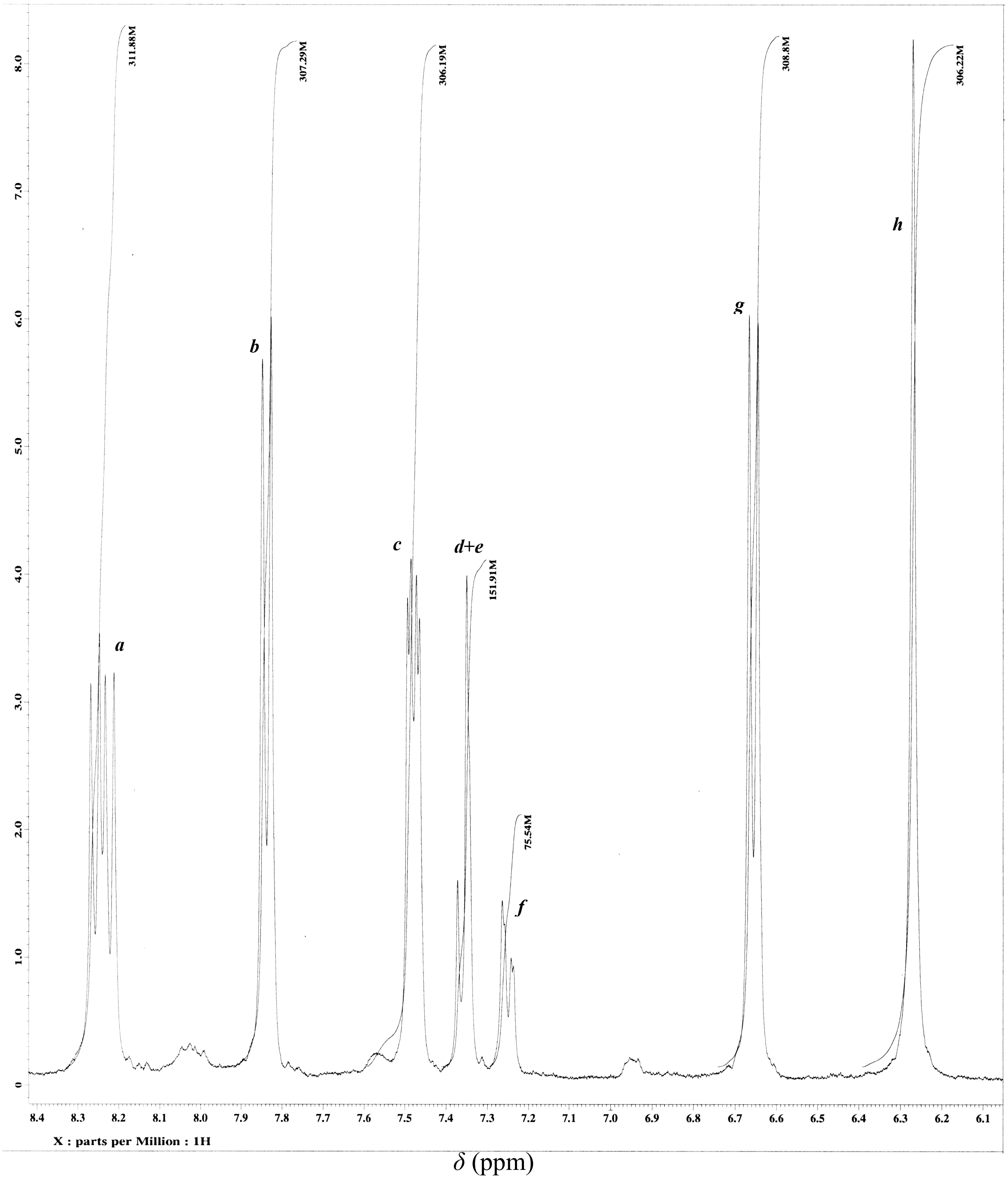
a
b
c
d+e
f
g
h
311.88M
307.29M
306.19M
151.91M
75.54M
308.8M
306.22M
0
1.0
2.0
3.0
4.0
5.0
6.0
7.0
8.0
8.4 8.3 8.2 8.1 8.0 7.9 7.8 7.7 7.6 7.5 7.4 7.3 7.2 7.1 7.0 6.9 6.8 6.7 6.6 6.5 6.4 6.3 6.2 6.1
X : parts per Million : 1H
δ (ppm)

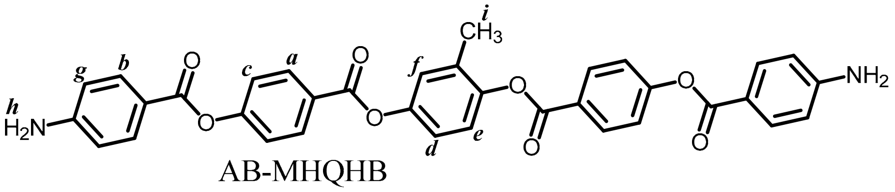
h
g
b
c
a
f
i
CH3
d
e
H2N
NH2
O

AB-MHQHB
